# Targeting Metabolism to Induce Cell Death in Cancer Cells and Cancer Stem Cells

**DOI:** 10.1155/2013/805975

**Published:** 2013-02-12

**Authors:** Claire Pecqueur, Lisa Oliver, Kristell Oizel, Lisenn Lalier, François M. Vallette

**Affiliations:** ^1^Equipe Labellisée Ligue Contre le Cancer, Equipe 9 CRCNA, INSERM UMR 892, CNRS UMR 6299, 44007 Nantes, France; ^2^Faculté de Médecine, Université de Nantes, 44007 Nantes, France; ^3^Centre de Recherche en Cancérologie Nantes Angers, INSERM UMR 892, CNRS UMR 6299, Université de Nantes 8 Quai Moncousu BP 70721, 44007 Cedex 1 Nantes, France; ^4^Centre Hospitalier, Universitaire (CHU) de Nantes, 44007 Nantes, France; ^5^Institut de Cancérologie de l'Ouest, René Gauducheau, Saint-Herblain, 44805 Nantes, France

## Abstract

Abnormal metabolism and the evasion of apoptosis are considered hallmarks of cancers. Accumulating evidence shows that cancer stem cells are key drivers of tumor formation, progression, and recurrence. A successful therapy must therefore eliminate these cells known to be highly resistant to apoptosis. In this paper, we describe the metabolic changes as well as the mechanisms of resistance to apoptosis occurring in cancer cells and cancer stem cells, underlying the connection between these two processes.

## 1. Introduction

Cell proliferation involves the replication of all cellular contents with the required energy for this to happen. In normal cells, glucose participates in cellular energy production through glycolysis as well as through its complete catabolism via the tricarboxylic acid (TCA) cycle and oxidative phosphorylation (OXPHOS). In addition to glucose, glutamine is also required to feed the TCA cycle. Lipids, amino acids, and nucleotides necessary for the biosynthesis of the daughter cells are mostly provided by intermediate metabolites of these pathways. To prevent aberrant cell proliferation, these pathways are tightly regulated. However, cancer cells overcome these controls, in particular by acquiring genetic mutations leading to the activation of oncogenes (pten, myc) or loss of tumor suppressors (p53) [1]. For example, a major regulator of metabolism is phosphoinositol 3 kinase (PI3K). PI3K is activated by growth factors resulting in, among others, the activation of Akt and mTOR. This activation is necessary for both cell proliferation as well as glucose uptake and use. In addition to its role in glucose metabolism, this pathway also regulates the redirection of free amino acids to protein synthesis via the mTOR-signaling pathway. 

## 2. Metabolic Modifications in Cancer Cells

In contrast to normal cells, most cancer cells predominantly produce energy by a high rate of glycolysis followed by lactate fermentation, even in the presence of oxygen, a less efficient metabolism compared to a low rate of glycolysis followed by mitochondrial oxidation of pyruvate [2]. Typically, rapidly proliferating tumor cells have glycolytic rates up to 200 times higher than those of their normal tissue of origin, even in the presence of oxygen [3]. This observation resulted in the development of 2-[18F]-fluoro-2-deoxy-D-glucose positron emission tomography (PET) to detect glucose uptake and lactate production for tumor imaging. 

**Figure 1:**
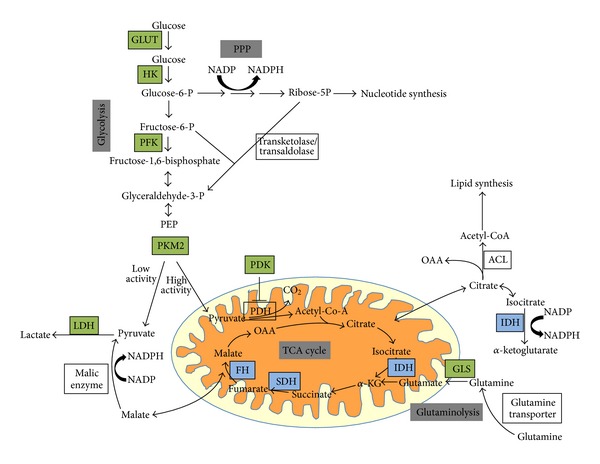
Metabolic adaptations of cancer cells. Glucose and glutamine are the 2 major substrates used by cancer cells. Glucose is imported into the cells through glucose transporters (GLUT) where it is phosphorylated by Hexokinase (HK). It will then be either metabolized through glycolysis or diverted to the pentose phosphate pathway (PPP). Glucose-derived pyruvate is mainly converted into lactate in cancer cells instead of being imported into mitochondria to be oxidized in acetyl CoA to support mitochondrial energy production. MYC enables cancer cells to maximize glutamine uptake from the extracellular space through the upregulation of the glutamine transporter. Once glutamine enters the cell, it can be metabolized through glutaminolysis to provide glutamate. The transamination of glutamate to *α*KG will feed the TCA cycle (adapted from Vander-Heiden et al. [9]). *α*KG: *α*-KetoGlutarate; TCA: tricarboxylic acid cycle; PDH: pyruvate dehydrogenase; LDH: lactate dehydrogenase; PDK: PDH-kinase; PK: pyruvate kinase; PEP: phosphoenolpyruvate; GLS: glutamine synthase; SDH: succinate dehydrogenase; FH: fumarate hydratase; 2-HG: 2D-hydroxyGlutarate; IDH: isocitrate dehydrogenase; HK: hexokinase; GAPDH: glyceraldehyde 3-phosphate dehydrogenase.

Pyruvate, which is at the crossroad between lactate production and OXPHOS, constitutes a key metabolic intermediate. In normal cells, the fate of pyruvate depends on many factors, one of which is oxygen availability. In the presence of oxygen, the pyruvate is directed into mitochondria to be converted into acetyl CoA by the pyruvate dehydrogenase (PDH) or into alanine by transamination. Inside the mitochondria, pyruvate is completely oxidized through the TCA cycle, feeding reductive equivalents to the electron transport chain. When oxygen is limited, as in muscles that have undergone prolonged exercise, pyruvate is not consumed in the TCA cycle but is rather converted into lactic acid by lactate dehydrogenase (LDH) in a process termed anaerobic glycolysis. In contrast, cancer cells shift their metabolism toward lactate production even in the presence of oxygen [4], partly through genetic modifications that stabilize the transcription factor Hypoxia Inducible Factor (HIF) involved in the adaptation of the cells to hypoxia, under nonhypoxic conditions as well as generating an adaptive response to the hypoxic microenvironment (Figure 1). By stimulating the expression of glucose transporters and glycolytic enzymes, HIF-1 promotes glycolysis to generate more pyruvate [5]. Furthermore, HIF-1 actively limits the mitochondrial consumption of pyruvate at two levels: (i) through the enzyme PDK (PDH-kinase), which in turn inhibits PDH activity preventing the conversion of pyruvate into acetyl CoA, and thereby limiting mitochondrial metabolism of pyruvate [6] and (ii) through the direct activation of LDH [7, 8]. Overall, these processes allow the regeneration of NAD^+^ required for ATP production through glycolysis.

Pyruvate synthesis by pyruvate kinase (PK) is modified in cancer cells. This step is highly regulated by the type of isoform expressed and/or by allosteric regulation. Four isoforms have been described with a specific tissue distribution. PKL is found in the liver and kidney and PKR in red blood cells. The two isoforms PKM1 and PKM2 are different splicing products of the same gene [10]. PKM1 is expressed in organs with high energy demands such as muscle and brain while PKM2 is expressed in differentiated tissue such as lung, fat, and pancreatic islets as well as in all cells with a high rate of nucleic acid synthesis such as proliferating cells, embryonic cells, and, especially, tumor cells [11]. In contrast to PKM1, which exists in a constitutively tetrameric active form, PKM2 exists under dimeric and tetrameric forms. The dimeric PKM2, which is inactive, results in an accumulation of upstream glycolytic intermediates, thus favoring their redistribution towards other biosynthetic pathways (synthesis of nucleic acids, phospholipids, or amino acids). PKM2 is different from other pyruvate kinase isoforms because it can bind to proteins that are phosphorylated on tyrosine residues in response to cell growth signals. This phosphotyrosine-binding activity negatively regulates the enzymatic activity providing a link between cell growth signals and the regulation of glycolysis. Thus, the ratio tetramer: dimer of PKM2 determines whether carbons from glucose are converted into lactate via pyruvate or channeled into building block synthesis (Figure 1). This ratio depends mainly on the availability of fructose-1,6-phosphate (FBP) since high concentrations of this enzyme induce the association of dimeric forms into tetramers, which in turn leads to lactate production with energy regeneration (Warburg effect) until the level of FBP is reduced and the tetramer dissociates into dimers.

Besides glycolysis, another metabolic pathway used by cancer cells to provide macromolecules is glutaminolysis, which generates reductive equivalents such as NADPH by replenishing the TCA cycle [4]. Glutamine is a conditional amino acid in the sense that, under normal conditions, it can be synthesized in most cells. However, during rapid growth, the cellular demand exceeds its supply and glutamine becomes essential. Glutamine provides energy through the TCA cycle as well as nitrogen, sulfur, and carbon skeletons for proliferating cells. Tumor cells tend to have a large pool of glutamate, and this pool is maintained by their ability to convert glutamine into glutamate through glutamine synthase (GLS), a mitochondrial enzyme highly active in tumors (Figure 1). In fact, limiting GLS activity results in a decreased growth rate in tumor cells both *in vitro* and *in vivo* [12]. Glutamate is also a nitrogen carrier for alanine and aspartate synthesis through the activity of aminotransferase. Alanine is used in protein synthesis and is also avidly secreted by tumor cells while aspartate contributes to the synthesis of proteins and nucleotides as well as feeding the electron transport chain via the malate/aspartate shuttle. Glutamine is also involved in the biosynthesis of glutathione, one of the major antioxidant molecules of the cells. In tumors, maintaining the reduced form of glutathione is crucial for cell survival since it allows the cell to resist oxidative stress associated with a rapid metabolism, DNA-damaging agents, or inflammation [13]. 

Beyond its roles in intermediary metabolism, glutamine exerts other effects that support cell survival and growth [10, 14]. Reflecting the importance of glutamine in anabolic metabolism, cells have developed glutamine-dependent mechanisms to control growth, including the modulation of signal transduction pathways. For example, a recent study showed that cellular uptake of glutamine and its subsequent rapid efflux in the presence of essential amino acids is required for the activation of the mTOR pathway [15]. Glutamine uptake is regulated by SLC1A5, and its loss inhibits cell growth and activates autophagy. Other data have identified a role for glutamine in the extracellular signal-regulated protein kinase- (ERK-) signaling pathway. This has been best characterized in intestinal epithelial cells, which consume glutamine as their major bioenergetic substrate and require glutamine for both proliferation and survival. In these cells, the addition of glutamine was sufficient to stimulate ERK signaling whereas glutamine deprivation was associated with increased apoptosis [16].

The interplay between glutamine and glucose utilization would depend on the particular oncogene/tumor suppressor involved in tumor progression. While the myc oncogene induces both aerobic glycolysis and glutaminolysis, activated *β*-catenin induces glutamine synthesis. However, glutamine synthetase is not highly expressed in all tissues, and thus glutamine consumption and addiction are dependent on the metabolic profile of the cancer cells. In addition, it has been postulated that ammonia, a byproduct of glutamine metabolism, is a diffusible activator of autophagy [17].

Finally, the tumor microenvironment containing supporting host cells (stroma, adipocyte, fibroblasts, muscle, and endothelial cells) and immune cells plays an important role in tumor initiation, tumor progression, and in the response of tumoral cells to therapy. Several recent publications have highlighted a metabolic crosstalk called the “reverse Warburg effect” where aerobic glycolysis in host stromal cells fuels anaplerotic metabolism in tumor cells [18]. In this two-compartment model, the anabolic tumor cells obtain energy from the surrounding host cells by inducing catabolic processes such as autophagy, mitophagy, and aerobic glycolysis, which would result in the overproduction of high-energy metabolites such as L-lactate, ketone bodies, and glutamine [19]. These metabolites are taken up by tumor cells and converted into acetyl CoA, which enters the TCA cycle resulting in the production of ATP. In addition, as a result of the enhanced glycolytic metabolism in the tumor, lactate accumulates in the tumor microenvironment. Besides the role of lactate in metastases [20, 21], this acidification plays an important role in tumor immunosuppression since lactate has been shown to inhibit the differentiation and/or the function of immune cells [22, 23]. 

## 3. Genetic Mutations in Metabolic Enzymes and Cancer

Although most cancer cells have functional mitochondria, a subset of human tumors harbors mutations that impair mitochondrial metabolism [24]. Two major classes of mutations occur in genes required for the function of the TCA cycle enzymes namely the succinate dehydrogenase (SDHA, SDHB, SDHC, SDHD, and SDHAF2) and the fumarate hydratase (*FH*) [25, 26]. Heterozygous germline mutations in SDHB, SDHC, and SDHD subunits were identified in paragangliomas and pheochromocytomas whereas germline mutations in FH predispose to renal cell cancer. In all cases, the loss of function mutations are followed by a somatic “second hit” resulting in the loss of the other allele in tumor cells. These mitochondrial enzymes catalyze, respectively, the conversion of succinate into fumarate and the reversible conversion of fumarate into malate in the TCA cycle (Figure 1). The loss of function of SDH and FH results in the accumulation of succinate and fumarate in the cytosol. The accumulation of succinate and fumarate impairs the enzymatic activity of several *α*KG-dependent dioxygenases including the PHDs (Prolyl-Hydroxylases). These proteins which are regulated by changes in the oxygen concentration initiates the hydroxylation of HIF-1*α* resulting in the ubiquitination /degradation of the *α*-subunit of HIF 1 under normoxia. However, only PHD2 has been shown to directly interact with HIF-1*α*. Similarly, fumarate inhibits PHD-2 activity leading to HIF-1*α* stabilization. A recent analysis shows that 12% of glioblastoma multiforme (GBMs), the most common and most aggressive malignant brain tumor, have a mutation in the gene-encoding isocitrate dehydrogenase-1 (IDH-1) [27]. This mutation is present in more than 90% of recurrent GBMs while it is present in less than 5% of *de novo* GBMs [28]. Mutations in IDH-1 and IDH-2 (isocitrate dehydrogenase-2) have also been identified in acute myeloid leukemia [29]. These enzymes catalyze the conversion of isocitrate into *α*KG and as such play important roles in metabolism and growth (Figure 1). IDH mutations are associated with a neomorphic activity of the enzyme leading to the production of an oncometabolite, 2D-hydroxyglutarate (2-HG) [30]. 2-HG accumulation impairs DNA methylation via the inhibition of *α*KG-dependent dioxygenases that carry out diverse functions such as prolyl-hydroxylation, histone demethylation, and epigenetic modifications of DNA [31, 32]. The expression of this mutation also impairs hematopoietic and adipocyte differentiation [33–35]. 

Finally, a common cellular response to impaired mitochondrial metabolism, for example, in cells deficient in FH, is the glutamine-dependent reductive carboxylation [36]. During this process, *α*KG is carboxylated by IDH isoforms to generate isocitrate, which in turn generates citrate, oxaloacetate (OAA), and acetyl CoA. The latter is crucial for fatty acids synthesis and protein acetylation while OAA is reduced to malate [36, 37]. This mechanism would enable cells with an impaired OXPHOS to maintain cell proliferation.

## 4. Metabolic Modification in Cancer Stem Cells 

The cancer stem cell concept was proposed several decades ago to explain two recurring observations. First, most cancers consist of phenotypically heterogeneous tumor cells, and, second, only a fraction of cells from both hematologic and solid tumors are tumorigenic [38–40]. Later, it was established that the tumorigenic potential was not equally shared by all cells within an individual tumor but restricted to a distinct subset. Thus, tumors are made up of a large subset of cells with a high rate of division unable to give rise to a new tumor and a small number of cells with a slow rate of division supplying the tumor with new tumor initiating cells. Two models could explain this tumor heterogeneity. The stochastic model predicts that tumors are biologically homogeneous and the behavior of the cancer cells is influenced by intrinsic or extrinsic factors resulting in a heterogeneity in the expression of cell markers, cell cycle, or in tumor initiation ability. In contrast, the hierarchic model predicts that tumors are organized as a normal tissue with stem cells maintaining the tissue hierachy [41, 42]. These CSCs were identified for the first time in acute myeloid leukemia [43]. They were described as an unusual and small population of cells (0.01–1% of the total population), capable of inducing leukemia after serial transplantation into immunodeficient mice. CSCs were subsequently identified in numerous solid tumors. In breast tumors, a population of cells enriched in markers CD44^+^ CD24^−/low^ was identified as CSCs [44]. More recently, CSCs have been described in brain tumors [45], medulloblastoma ependymoma [46], colorectal tumors [47], pancreas [48], ovarian [49], liver [50], prostate [51], lung [52], and in melanomas [53]. Like normal stem cells, CSCs reside in niches, that is, a microenvironment capable of maintaining a balance between self-renewal and differentiation. However, all tumors do not seem to follow the model suggested by the presence of CSCs. Indeed, some tumors have little heterogeneity and seem to follow a model of clonal evolution or a stochastic model, in which a population of proliferating cells gives rise to the tumor [54]. Nevertheless, both models are not mutually exclusive. Indeed, the CSCs may undergo clonal evolution and become more aggressive due to mutations or epigenetic modifications. This phenomenon has been described in leukemia [55] and has also been observed in the case of serial transplantation in animals, which generate more aggressive tumors [56]. Finally, a controversy exists about whether these CSCs are derived from normal stem cells that have transformed or cancer cells that have dedifferentiated. 

At present, we do not know if CSCs come from normal stem cells or more differentiated cells that have acquired dedifferentiating mutations. One hypothesis for the existence of CSCs suggests that these cells derive from normal stem cells that have acquired mutations that allow them to escape the control of the niche. Another hypothesis is that dysregulation of growth factors secreted by the niche could lead to uncontrolled proliferation of stem cells and, as a result, tumorigenesis [57].

Cancer stem cells have been defined, by analogy to normal stem cells, in that they are capable of self-renewal and can generate all the differentiated cells found within the tumor [45, 58]. One feature of CSCs is their ability to expel chemicals, most often lipophilic, via membrane transporters (multidrug resistance proteins: MDR). Thus, in cancer cell cultures grown in the presence of Hoechst 33342 (a DNA intercalant), a portion of the cells, called side population (SP), remains unlabelled and may be isolated on this basis. This population (0.15 to 1.2% of the total population) has characteristics of CSCs, that is, the ability to form neurospheres when cultured in defined medium over a long term (self-renewal) while retaining the ability to differentiate into neurons and glial cells at each passage (multipotency) and the ability to trigger tumor formation after injection into immunocompromised mice [59, 60]. Several surface markers are currently used to identify CSCs. As cited before, CSCs are identified as CD44^+^  CD24^−/low^ in breast tumors [44] while, in gliomas, they have been identified mostly on the basis of the expression of CD133 (or prominin 1) [45]. However, the CD133 marker has been questioned since it is a target gene of HIF-1, one of the main transcription factors of hypoxia [61], and its expression can be increased by chemical or genetic dysfunction of mitochondria [62]. Thus, at least for gliomas, the identification of other CSCs markers, such as nestin, an intermediate filament, a marker of neural stem cells [63], or CD15 (SSEA1 or Lewis X) present in primary neurospheres [64], is under evaluation. Finally, if different surface markers have been described for CSCs from various tumors, most of these markers are shared with normal stem cells.

Normal mouse embryonic stem cells (ESCs) exhibit a bivalent metabolism (glycolytic or phosphorylative depending on the cell requirements). However, human ESCs exhibit a glycolytic metabolism, probably due to defective mitochondria [65]. Based on these studies or those in the early stage of embryos [66, 67], highly undifferentiated cells such as CSCs should be able to revert between aerobic glycolysis and glutaminolysis. Several studies have shown a glycolytic phenotype in CSCs with an overexpression of most glycolytic enzymes (Figure 1) [68, 69]. Several isoforms of LDH, known to be upregulated under hypoxia as well as c-MYC, are commonly highly expressed in CSCs, which facilitate the diversion of glucose carbons away from oxidative metabolism. However, in a similar way that some cancer cells exhibit an oxidative rather than glycolytic metabolism, CSCs have different metabolic profiles depending on their tissue of origin and their degree of differentiation. For example, highly undifferentiated liver cancers tend to be more glycolytic than tumor cells that retain some differentiation characteristics [70]. A recent study using a glioma stem cells model showed that these cells consumed less glucose and produced less lactate compared to their cancer cell counterparts [71]. 

Serine and glycine are both nonessential amino acids that can be taken up by cells or synthesized from 3-phosphoglycerate. These amino acids are important precursors for nucleotide and glutathione synthesis. In CSCs isolated from nonsmall cell lung cancer, Zhang et al. showed a high upregulation of genes involved in serine and glycine metabolism concomitant with an upregulation of glycolytic genes [72]. In particular, the expression of the glycine decarboxylase (GLDC) was markedly upregulated in these CSCs. These authors also showed that GLDC overexpression alone was able to transform NIH 3T3 cells *in vitro* and drive tumor formation *in vivo*, while silencing of this enzyme-diminished tumorigenicity.

In addition to the intrinsic needs of the cells, exogenous factors influence both cellular fate and metabolic processes. The resident microenvironment, also known as the niche, is an indispensable factor that distinguishes normal stem cells from CSCs. The niche is the source of molecules that activate or inhibit signal transduction pathways. While the stem cell microenvironment of a normal tissue is known to maintain a balance between self-renewal and differentiation [73–75], the tumor microenvironment required for the maintenance of CSCs is altered, retaining predominantly proproliferating signal [76, 77]. The role of the tumor microenvironment in tumor initiation and progression through stromal cells or immune cells, as well as alterations in extracellular modeling or oxygen concentration, is widely accepted [78]. These niches are characterized by a low oxygen concentration and as such promote a glycolytic phenotype mediated, in part, through the HIF-signaling pathway. During cancer initiation, a hypoxic environment would favor the activation of genes associated with “stemness” such as Notch or Oct4 as well as genes associated with the glycolytic switch, for example glucose transporters, (hexokinase) HK, PKM2, LDH, and PDK [79]. In fact, increased expression of nestin through the activation of the Notch-signaling pathway has been detected in glioma cell lines [80]. The capacity of CSCs to modulate the tumor microenvironment has also been suggested. In solid tumors, the adaptation of CSCs hypoxia resulting in a glycolytic shift would mediate the acidification of the tumor microenvironment. In fact, local pH measurements revealed a shift from 7.1 in normal brain tissue to 6.8 in brain tumors with some being as low as 5.9 [81]. This acidification promotes the maintenance of the stem cell phenotype. In addition, microenvironment acidification would in turn alter the activity of proteases that are implicated in the degradation of the extracellular matrix. In fact, several studies have shown that hypoxia promotes metastasis through HIF-dependent pathways [82] and through the activation of enzymes involved in the rigidity of the extracellular matrix such as lysyl oxidases [83]. On the clinical level, there is a direct correlation between the presence of a hypoxic core within the tumor and a poor prognosis for patients [82, 84, 85].

## 5. Resistance to Therapy of Cancer Cells

**Figure 2:**
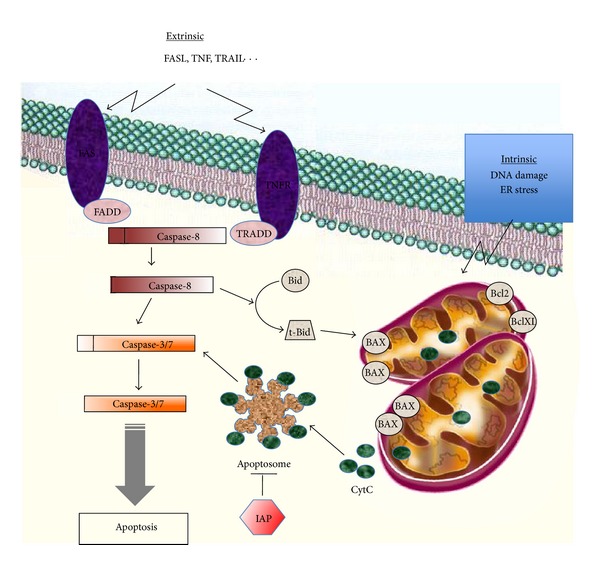
Intrinsic and extrinsic apoptotic pathways. Apoptosis can be triggered by the intrinsic mitochondrial pathway or through the extrinsic pathway involving the death receptors. The intrinsic apoptotic pathway is activated in response to various stimuli such as DNA damage, endoplasmic reticulum (ER) stress, or hypoxia. This pathway is mainly modulated through differential interactions between the antiapoptotic (Bcl-2, Bcl-XL), the proapoptotic (Bax, Bak), and the BH3-only proteins (Bad, Bid, Bim…). Bax, Bid, and Bim are initially inactive and must translocate to the mitochondria to induce apoptosis, either by binding via BH3 domains to Bcl-2, Bcl-XL and antagonizing their antiapoptotic functions or through the permeabilization of the mitochondrial membrane. Permeabilization of the mitochondrial membrane releases apoptogenic proteins, among which the cytochrome c is leading to the formation of the apoptosome, activation of caspase 9, and ultimately to the activation of effector caspases. In the extrinsic pathway, ligands (TNF, FASL, or TRAIL) bind to their specific death receptors, which lead to their oligomerization, recruitment of procaspase 8, and a specific adaptor protein (FADD and TRADD). The formation of the DISC induces autocatalysis of procaspase 8 into its active form, which in turn leads to the activation of the effector caspases.

Evasion of programmed cell death or apoptosis has been recognized as one of the main alterations that dictate malignant growth and is a hallmark of most types of cancer [86]. Apoptosis can be triggered either by the intrinsic (mitochondrial) pathway or the extrinsic (death receptor) pathway. The central players in both pathways are the family of caspases (Figure 2). The activation of the intrinsic pathway induces mitochondrial membrane permeabilization leading to the release of apoptogenic proteins including cytochrome c, and ultimately to the activation of caspase cascade, DNA fragmentation, and cell death. The BCL-2 family of proteins, consisting of antiapoptotic, proapoptotic, and BH3-only proteins, plays a central role in controlling the intrinsic pathway. These proteins are located or translocated to the mitochondrial membrane and modulate apoptosis by altering the outer mitochondrial membrane permeability [87]. The extrinsic pathway is activated through the (tumor necrosis factor) TNF receptors. After binding of its ligand (TNF*α*, FAS-L, or TRAIL), the receptor oligomerizes, leading to the formation of the (death-inducing signaling complex) DISC (death-inducing signaling complex) with the recruitment of a specific adaptor protein leading ultimately to the activation of caspase 8. DISC activation will either directly activate effector caspases or cleave the BH3-only protein Bid, which in turn would engage the mitochondrial pathway through the activation of proapoptotic Bax. Apoptosis is also controlled by the Inhibitors of Apoptosis Proteins (IAPs), including survivin, and the FLIP proteins that inhibit the activation of caspase-8. 

Antiapoptotic pathways are generally enhanced in tumor cells, which promote their survival but also render the cells more dependent on antiapoptotic pathways thus providing a potential therapeutic window. The intrinsic pathway of apoptosis is activated in the presence of most anticancer drugs and other stresses such as growth factor deprivation or DNA damage [75, 76]. It has been demonstrated that resistance to chemotherapy-induced apoptosis in several tumor cell types is controlled by antiapoptotic proteins Bcl-2, or Bcl-X_L_ while sensitivity to apoptosis *in vivo* is associated with increased levels of Bax [88]. Recently, it has been shown that Bcl-X_L_ protects against apoptosis through a mechanism independent of proapoptotic proteins Bax/Bak, by reducing glucose-derived citrate, which in turn caused a decrease in the levels of acetyl CoA and protein N-alpha acetylation [89]. The latter would affect protein activity, stability, assembly, and localization within the cell. 

The activation of the extrinsic pathway promotes apoptosis in many types of tumors. However, toxic side-effects were observed with recombinant TNF and agonistic anti-FAS antibodies limiting their therapeutic use [90]. A potentially more promising approach involves targeting the TRAIL receptors. Phase I clinical trials have established the safety and tolerability of these TRAIL agonists in patients [91]. Phase II trials are currently evaluating the therapeutic efficacy of TRAIL agonists as single agents or in combination with established cancer therapy. Unfortunately, about 50% of cancer cell lines are resistant towards TRAIL-induced apoptosis [92] and furthermore, TRAIL receptors can elicit prosurvival or proinvasive effects, both of which are counterproductive in treatment [93]. Finally, some recent studies have shown that cell death resistance could be linked to alterations in the structure of mitochondria [83, 84]. 

## 6. Mechanism of Cell Death Resistance in CSCs

Cancer stem cells being more quiescent are more resistant to apoptosis. The role of Bcl-2 in protecting hematopoietic CSCs against apoptosis has been demonstrated both *in vitro* and *in vivo* [94] as well as in response to radiation [95]. Similarly, Bax^−/−^ mice exhibit increased multipotent progenitor cells [96]. In gliomas, antiapoptotic genes, including *flip*, *bcl-2,* and *bcl-xl*, as well as IAP family members (*xiap, ciap1, ciap2, naip,* and *survivin*) are found at higher levels in CSCs (CD133^+^), and this correlates with enhanced drug resistance to different agents including temozolomide, carboplatin, VP16, and Taxol [88]. Furthermore, a high Mcl-1 expression was associated with resistance to ABT-737, a BH3-mimetic, in glioma stem cells [97].

Resistance to cell death upon radio- or chemotherapy is also mediated through the DNA damage repair (DDR) machinery. In fact, glioma CSCs exhibit a higher capacity of DDR mediated, to some extent, through an elevated activation of checkpoint kinases Chk1 and Chk2, in response to radiation [98]. Furthermore, the DNA repair enzyme MGMT (O6-methylguanine-methyltransferase) is usually overexpressed in these cells [88]. In fact, several studies have shown that MGMT overexpression predicts a patient response to temozolomide, an alkylating drug that prolongs survival when administered during and after radiotherapy in first-line treatment in GBM [99]. Similar studies in mesenchymal stem cells (MSCs) have highlighted the tumor-promoting role of p21 [100]. The transformation of these cells has been to some extent associated with deregulation of the cell-cycle proteins p16 and CDK triggering an increase in resistance to apoptosis [101]. The activation of DDR through p21 appears to be implicated in leukemia CSCs self-renewal [102]. Interestingly, human MSCs are resistant to apoptosis when undifferentiated but become sensitive to cell death upon the initiation of differentiation [103]. 

Glioma CSCs are also resistant to TRAIL-induced apoptosis partially through methylation of caspase 8 [104] although it was suggested that this resistance could be overcome by treating with the proteasome inhibitor bortezomib [105] or with cisplatin in breast CSCs [106]. Sensitivity to TRAIL-induced apoptosis is increased in colon CSCs, defined as the SP by Hoechst 33342 staining. These cells, known to be resistant to chemotherapy, express higher levels of TRAIL-receptor 1 (TRAIL-R1) that correlates with increased sensitivity to TRAIL-induced apoptosis [107]. 

Finally, the efficiency of apoptosis targeting agents is limited by the presence in CSCs of active transmembrane ATP-binding cassette (ABC) transporters involved in the efflux of drugs. For example, enhanced resistance of glioma CSCs (CD133^+^) to temozolomide or etoposide is mediated by a higher expression of ABCG2 [88]. Breast cancer cells and isolated mammary gland CSCs are also less sensitive to treatment through increased activity of Wnt pathway leading to an overexpression of MDR1 [108]. Current hypotheses suggest that this CSCs resistance to treatment could also be mediated through the protection accorded by the niche. 

## 7. Impact of Metabolism on Cell Death

We have illustrated above an increasing amount of evidence suggesting that metabolic alterations are primary events in the transformation process, whether this is through activation of oncogenes, inactivation of tumor suppressors, or mutations in genes encoding metabolic enzymes. However, how specific metabolites contribute to apoptosis in tumor cells remains a central question. One possible link between metabolic change and resistance to apoptosis is the association of HKs with the voltage-dependent channel protein (VDAC) under glycolytic metabolism. While this interaction facilitates the phosphorylation of glucose using ATP generated by mitochondria, it also prevents the binding of proapoptotic proteins such as Bak with VDAC, thereby preventing apoptosis [96, 97]. Another glycolytic enzyme with a proapoptotic function is Glyceraldehyde 3-Phosphate Dehydrogenase (GAPDH), which translocates to the nucleus in cultured neurons and induces neuronal death when over-expressed [109]. The fact that GAPDH is over-expressed in cancer cells seems paradoxical since it was shown that GAPDH is able to prevent caspase-independent cell death by increasing the amount of intracellular ATP and by stimulating autophagy [99, 100]. 

Tp53-Induced Glycolysis and Apoptosis Regulator (TIGAR), a target of p53, inhibits glycolysis by reducing the level of FBP. Glucose is then redirected into the pentose phosphate pathway (PPP) to produce NADH and nucleotides instigating an increase in glutathione. As such, TIGAR decreases the sensitivity of cells to p53 and other apoptotic signals associated with ROS [101, 102]. Similarly, an over-expression of PFK diverts glucose from glycolysis to the PPP and increases the resistance to oxidative stress [110].

Attempts have been made to modulate metabolic reprogramming by treating with compounds that inhibit glycolysis. Several studies show that glucose deprivation leads to cell death. For example, glioma cells cultured in the absence of glucose die by ROS-induced apoptosis suggesting that, in the absence of glucose, these cells are able to change their metabolism and use their mitochondria to produce ATP [111]. Another study showed that a shortage of glucose would induce cell death in cells deficient in Bax and Bak, effectors of mitochondrial permeabilization via an unconventional pathway requiring caspase 8 [112]. This effect is highlighted using the glucose analog, 2-deoxyglucose (2-DG), which accumulates in cells and inhibits HK. At high concentrations, 2-DG causes a decrease in ATP levels resulting in cell death, especially in cells with mitochondrial defects or under hypoxia [113]. This compound has entered numerous clinical trials in combination with other agents and seems to potentiate the effect of radiotherapy, at least in patients with brain tumors [114]. Dichloroacetate (DCA) is another molecule involved in the downregulation of glycolysis. This small molecule inhibits mitochondrial PDK, forcing pyruvate into the mitochondria thereby increasing mitochondrial metabolism [115]. Indeed, DCA decreases tumor growth *in vitro* and *in vivo* without affecting normal tissue [116–118]. While DCA alone has no effect on apoptosis in glioma CSCs, it induces a Bax-dependent apoptosis in these cells when combined with etoposide or radiation [69]. Recently, it was shown that DCA, already used in the clinical treatment of genetic mitochondrial diseases [119], could be used in patients with GBM by inhibiting PDK over-expressed in these tumors [120]. One of the main properties of this molecule is its blood-brain barrier permeability. Furthermore, a direct consequence of downregulating glycolysis by DCA is an increase in intracellular pH, which in turn decreases the invasive ability of tumors. 

PKM2 is a promising target for potential therapeutic approaches since the ratio of PKM2 tetramer: dimer has severe consequences on metabolism, proliferation and the tumorigenic capacity of the cells [121, 122]. Furthermore, this isoform can translocate into the nucleus where it can either induce cell death upon various apoptotic stimuli such as UV or H_2_O_2_ [123] or interact with transcription factors involved in the “stemness” such as Oct4 [121, 122]. Many inhibitors capable of blocking the allosteric regulation of the M2 isoform are currently under investigation. It was shown that a peptide (Aptamer 9) blocked PKM2 in its inactive conformation thereby decreasing cell proliferation even in the presence of high concentrations of glucose [124]. A recent study revealed new activators (diarylsulfonamides) of this enzyme, the effects of which are still to be demonstrated [125]. 

Differentiation therapy has also been exploited with Bone Morphogenetic Protein-4 (BMP4) treatment to induce glial differentiation reducing tumor growth in gliomas. Interestingly, after this treatment, glioma CSCs are unable to form tumors after transplantation in series in immunocompromised animals [126]. This study suggests a new treatment for GBM that would force the CSCs to enter differentiation, resulting in, firstly, a reduction in tumor mass and, secondly, a decrease in resistance to apoptosis of these cells.

## 8. Conclusion

**Figure 3:**
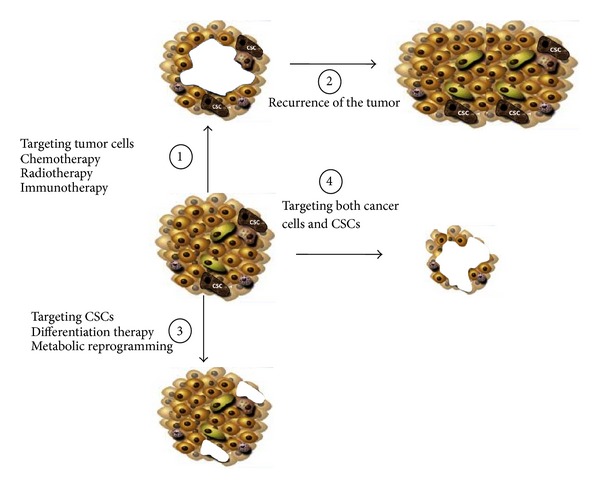
Needs of combinatorial therapies that target cancer cells and CSCs. A tumor is a complex mix of cancer cells including differentiated cells at different stages as well as CSCs. Current treatments kill cancer cells without affecting cancer stem cells (1). However, since CSCs are not affected, there is a major risk of tumor recurrence (2). Targeting only CSCs might result in a reduced number of matured cells but that will not be sufficient to eradicate the tumor (3). Thus, cancer therapy should ideally target both CSCs and matured cells (4) by generally increasing sensitivity to cell death of all the cell types as well as reducing the proliferation of matured cells and inducing differentiation and sensibility to cell death of CSCs.

The successful elimination of a cancer requires an anticancer therapy that will affect both differentiated cancer cells and CSCs (Figure 3). At present, conventional therapy that includes radio-, chemo-, and immunotherapy kills rapidly proliferating and differentiated cells. These treatments may cause the tumor to shrink but will not prevent tumor recurrence. Thus, a combination of treatments targeting both rapidly proliferating cancer cells and quiescent or slow-proliferating CSCs would be ideal. Therefore, it is essential to identify specific markers that distinguish between tumorigenic and nontumorigenic stem cells. These therapeutic strategies for CSCs include targeting pathways involved in the self-renewal process, differentiation, and “exit” from the niche. Furthermore, a reversal of tumor metabolism to “normal” might impair tumor growth of cancer cells, causing tumor regression, and differentiation/sensitization to cell death of CSCs, impairing the recurrence of the tumor.

## Abbreviations

Chk: Checkpoint kinase
DCA: DiChloroAcetate
2-DG: 2-DeoxyGlucose
*α*KG: *α*-KetoGlutarate
CSCs: Cancer stem cells
DISC: Death-inducing signaling complex;
DDR: DNA damage repair
FBP: Fructose-1,6-biPhosphate
FH: Fumarate hydratase
GBM: Glioblastoma multiforme
GLS: Glutamine synthase
GLUT: Glucose transporters
2-HG: 2D-hydroxyGlutarate
HK: Hexokinase
HIF: Hypoxia inducible factor
GAPDH: Glyceraldehyde 3-phosphate dehydrogenase
IDH: Isocitrate dehydrogenase
LDH: Lactate dehydrogenase
MGMT: O6-methylguanine-methyltransferase
MSCs: Mesenchymal stem cells
OAA: Oxaloacetate
OXPHOS: Oxidative phosphorylation
PPP: Pentose phosphate pathway
PI3K: Phospho-inositol 3 kinase (PI3K)
PHD-2: Prolyl hydroxylase domain protein-2
PDH: Pyruvate dehydrogenase
PDK: PDH-kinase;
PK: Pyruvate kinase
SDH: Succinate dehydrogenase
TCA: Tricarboxylic acid cycle.
